# What Causes the Dentin to Be Sensitive

**Published:** 1908-10-15

**Authors:** C. F. Kabell


### WHAT CAUSES THE DENTIN TO BE SENSITIVE.

BY C. F. KABELL, D.D.S.

The dentinal tubuli by the very minuteness of their size must have very strong capillary attraction, therefore, are necessarily filled with liquid, be it serum, nerve fluid, lymph or plain water.

In the healthy tooth these tubules will be entirely full, but through decay or operative measures, the exposed openings of the tubuli are subjected to atmospheric pressure, evaporation and the affinity of acids and alkali for water.

Let us first consider atmospheric pressure. If a capillary tube that has the capillary force to lift water ten feet is broken to the length of six inches, the water in this six inch tube will not run out of the opening but will sink a trifle in the tube, because the free opening is subjected to atmospheric pressure.

This will happen in the dentinal tubes when cut by the drill; the volume of liquid in the cut tubuli will sink and exert a pressure on the protoplasm of the odontoblasts which penetrates thread-like into the other end of the tubuli.

This sudden shock is transmitted as pain by the nerve fibrils which are supplied to the odontoblasts.

As proof of this theory I mention:

1st. The very painful sensation when we drill at right angles to the tubuli, because we constantly irritate nerve fibrils through the sinking of the liquid in the tubules.

2d. When we drill parallel to the tubuli the procedure is less painful because we administer a succession of shocks to the same nerve endings, which lose their transmission power on account of the physiological fact that nerves easily grow tired and in that state will not respond any more to irritation.

The removal of decalcified dentin is painful because before its removal it closed the tubuli and the excavating exposes the tubuli to free atmospherical pressure.

Hot air causes pain by evaporating the liquid out of the tubuli. This loss its capillary attraction will seek to supply from intercellular liquid in the pulp chamber.

Changes of temperature by hot or cold water or fillings cause expansion or contraction of the intertubular liquid with the resultant fluctuation in volume.

Spunk, I think, causes pain by absorbing some of the liquid out of the tubuli, although its action can probably be explained in the following way: When moisture is covering all the tubular openings, it will be the chief source of supply for the dentinal tubuli, so that atmospheric pressure will not be felt very much, in the same way as a stream of blood-warm water directed against the field of operation will ease the pain of drilling.

On removing this moisture, the intercellular liquid is again called upon with resultant disturbances and pain.

Alcohol, acids and alkalis act in a similar manner by their affinity for water, thereby causing a replenishing from the liquid in the pulp chamber.

Here osmosis might play an important role after the first shock of the application, to explain their sometimes desensitizing action.

### PROOFS OF THE THEORY.

In preparing a tooth for crowning, I found the dentin so sensitive to grinding that I had to devitalize. I was lucky to open the pulp chamber quite freely and here the idea struck me to test this theory.

I reasoned that if atmospheric pressure were now directly supplied to the contents of the pulp chamber, it would counterbalance any action of the same force directed to the tubuli, and sure enough, the dentin which before would not bear any grinding could now easily be cut without any pain to the patient.

Another proof is the action of nitrate of silver; by mechanically closing the tubuli it materially reduces the pain of cavity preparation.

In order now to understand why such minute changes in the amount and movement of the intertubular liquid will effectively cause shock and resultant pain, we must assume that the protoplasm of the odontoblasts snugly fills the opening of the pulp chamber ends of the tubuli, an assumption we have to take for granted when we consider pressure anesthesia.

When we apply a concentrated solution of cocain to the dentin, hydrolytic action will set in and the amount of the cocain in the intertubular liquid will soon be equal to the remaining amount of the salt in the applied solution, and if the tubuli were not closed the intercellular liquid would soon have an equal quantity of cocain throughout the pulp chamber.

As a simple topical application has no perceptable effect, this assumption must be correct, and only by applying pressure do we mechanically open the tubuli and gain the desired result.

This last sentence I leave open to discussion because I am not sure whether the sluggishness of a strong cocain solution will have any part in dissensitization or what factors outside of chemical action might contribute to the result, but in my humble opinion, I claim that this theory explains pretty well the different phenomena of dentinal sensation and the effects of our different methods to overcome it.—*Items of Interest.*

